# Morphometry and Stiffness of Red Blood Cells—Signatures of Neurodegenerative Diseases and Aging

**DOI:** 10.3390/ijms23010227

**Published:** 2021-12-25

**Authors:** Velichka Strijkova-Kenderova, Svetla Todinova, Tonya Andreeva, Desislava Bogdanova, Ariana Langari, Avgustina Danailova, Sashka Krumova, Elena Zlatareva, Nikolay Kalaydzhiev, Ivan Milanov, Stefka G. Taneva

**Affiliations:** 1 Institute of Biophysics and Biomedical Engineering, Bulgarian Academy of Sciences, “Acad. G. Bontchev” Str. 21, 1113 Sofia, Bulgaria; vily@iomt.bas.bg (V.S.-K.); todinova@abv.bg (S.T.); arianaiilias@abv.bg (A.L.); avgustina_danailova@abv.bg (A.D.); sakrumo@gmail.com (S.K.); 2 Institute of Optical Materials and Technologies “Acad. Yordan Malinovski”, Bulgarian Academy of Sciences, “Acad. G. Bontchev” Str. 109, 1113 Sofia, Bulgaria; 3 Faculty of Applied Chemistry, Reutlingen University, Alteburgstraße 150, D-72762 Reutlingen, Germany; 4 University Multiprofile Hospital for Active Treatment in Neurology and Psychiatry “St. Naum”, “Louben Roussev” Str. 1, 1113 Sofia, Bulgaria; dessislava_bogdanova@abv.bg (D.B.); el.zlatareva@gmail.com (E.Z.); kalaydzhiev_nikolay@abv.bg (N.K.); milanovivan@yahoo.com (I.M.)

**Keywords:** neurodegenerative disorders, red blood cells, cell morphology, surface roughness, diameter, height, volume, Young’s modulus, atomic force microscopy, optical microscopy

## Abstract

Human red blood cells (RBCs) are unique cells with the remarkable ability to deform, which is crucial for their oxygen transport function, and which can be significantly altered under pathophysiological conditions. Here we performed ultrastructural analysis of RBCs as a peripheral cell model, looking for specific signatures of the neurodegenerative pathologies (NDDs)—Parkinson’s disease (PD), amyotrophic lateral sclerosis (ALS) and Alzheimer’s disease (AD), utilizing atomic force (AFM) and conventional optical (OM) microscopy. We found significant differences in the morphology and stiffness of RBCs isolated from patients with the selected NDDs and those from healthy individuals. Neurodegenerative pathologies’ RBCs are characterized by a reduced abundance of biconcave discoid shape, lower surface roughness and a higher Young’s modulus, compared to healthy cells. Although reduced, the biconcave is still the predominant shape in ALS and AD cells, while the morphology of PD is dominated by crenate cells. The features of RBCs underwent a marked aging-induced transformation, which followed different aging pathways for NDDs and normal healthy states. It was found that the diameter, height and volume of the different cell shape types have different values for NDDs and healthy cells. Common and specific morphological signatures of the NDDs were identified.

## 1. Introduction

Neurodegenerative diseases (NDDs) are generally associated with various symptoms such as progressive degeneration and/or loss of neurons in the central and peripheral nervous system, problems with movement and/or mental functioning, i.e., with cognitive impairment and dementia. On the other hand, they are characterized by common underlying mechanisms in pathogenesis and progression, such as oxidative stress and generation of reactive oxygen species [1,2,3,4,5], mitochondrial dysfunction [6,7], dysregulation of calcium homeostasis [8,9,10,11], blood-brain barrier dysfunction [12], significant reduction in the expressions of Na+/K+-ATPase, α-spectrin and drebrin (involved in the function and integrity of neuronal membrane) [13], neuroinflammation [14,15,16], as well as by abnormal accumulation and misfolding of specific proteins, mainly β-amyloid peptide (Aβ), τ-protein and α-synuclein (α-syn), in the brain as well as in the peripheral blood cells, peripheral tissues and body fluids [4,17,18,19].

Despite advances in diagnostic and therapeutic methods, there is no cure for NDDs and the treatments used only alleviate symptoms or slow the progression of the disease. Developing new diagnostic approaches, respectively, the discovery of novel reliable, more cost-effective and readily accessible diagnostic biomarkers, as well as establishment of new therapies for these diseases are of extreme importance. The implication of cytokines and growth factors in neuroinflammation, and especially in AD [20], the therapeutic benefits of metformin as neuroprotective and anti-aging drug [[21] and references therein] and of medicinal plants for the treatment of neurological disorders [22] have been recently reviewed.

The interest in this investigation was triggered by a wealth of data in the literature on the existence of a tight but still unclear relationship between the structural features of red blood cells (RBCs) and NDDs. The structure of RBCs is maintained by a spectrin network imbedded in the lipid bilayer via ankyrins and binding complexes that determine the cells deformability [23,24,25]. The binding complexes contain the Band-3 protein, which plays a major role in the cell metabolism, the membrane integrity and the oxygen exchange between hemoglobin (Hb) and tissues [23,24,25]. RBCs membrane proteins, Band 3 protein (a major component of the membrane skeleton), Hsp90 and calpain-1 were suggested as promising preclinical biomarkers for cognitive impairment [26].

Being anuclear cells, RBCs are very sensitive to pathological conditions under which morphological transformations of the typical for healthy human cells biconcave discoid shape to atypical morphologies (spiculated cells (echinocytes and acanthocytes), spherocytes etc.) were found for several diseases [27,28,29]. Spiny RBCs, acanthocytes, are characteristic for hereditary neurodegenerative disorders (neuroacanthocytosis (chorea–acanthocytosis and X-linked McLeod syndrome)) and also occasionally in movement disorders such as Huntington’s disease-like 2 and pantothenate kinase-associated neurodegeneration [30,31]. Pretorius et al. [32] have shown that inflammatory signaling can induce damages in the morphology of RBCs (cell shrinkage and membrane blebbing) and apoptosis (eryptosis) in PD patients.

RBCs circulation in the blood stream exposes them to the influence of toxic pathological proteins (such as Aβ peptide, α-syn, τ-protein and their heterocomplexes) that propagate and accumulate in Alzheimer’s disease (AD), Parkinson’s disease (PD), and amyotrophic lateral sclerosis (ALS) [17,33,34,35]. Recent studies have shown that 98% of the peripheral RBCs from AD patients are able to bind amyloid peptides, while this percentage is only 38% for healthy individuals [36], suggesting a pathogenic role of the RBCs-amyloid peptides complexes. Moreover, Aβ binding to RBCs in the peripheral blood of patients with AD has been shown to modify the morphology of RBCs [36].

In PD, the total τ-protein concentrations in RBCs have been found to correlate with cognitive deficits in newly diagnosed patients [37,38] and α-syn (marker for sporadic PD) was found to be localized not only in the cerebrospinal fluid, but also in blood plasma, platelets and peripheral blood mononuclear cells. Although there are some contradictory results for the α-syn levels in peripheral fluids (plasma/sera) [39] and total α-syn in RBCs [40], higher oligomeric-α-syn concentration was found in PD patients compared to controls [41,42]. The levels of total α-syn, proteinase K-resistant (PKres) α-syn, phospho Serine 129 α-syn and oxidized α-syn were suggested as a complex biomarker for PD [43]. In addition, co-aggregates of α-syn with its mutational variants and with Aβ and τ-protein, were shown to be implicated in Alzheimer’s disease [44]. In fact, it is supposed that intact or lysed RBCs that have entered the cerebrospinal fluid are the source of α-syn [35]. Matsumoto et al. [45] recently demonstrated that accumulated α-syn is secreted by RBCs in the form of extracellular vesicles that cross the blood-brain barrier.

RBCs are thought to contribute also to the development of ALS through the secretion of damaging molecules. Indeed, a correlation was found between the progression of the disease and the increased activity of acetylcholine esterase, the increased erythrocyte deformability and the reduced flow of nitric oxide from RBCs [46].

This work is based on the hypothesis that the selected neurodegenerative pathologies cause specific changes in the surface roughness (R_rms_), stiffness and morphology (shape, diameter (D), height (H), volume (V)) of RBCs. We combined atomic force microscopy (AFM) and optical microscopy (OM) to establish specific morphometric features and alteration in the Young’s modulus (*E*) of RBCs from PD, ALS and AD patients, and their dependence on the cells age. We found considerable differences in the shape distribution, R_rms_, V and *E* of RBCs derived from patients with the three studied NDDs and from healthy individuals and suggested that they could be used as signatures for the studied pathologies.

## 2. Results

### 2.1. RBCs Morphology in Neurodegenerative Disorders

**Figure 1:**
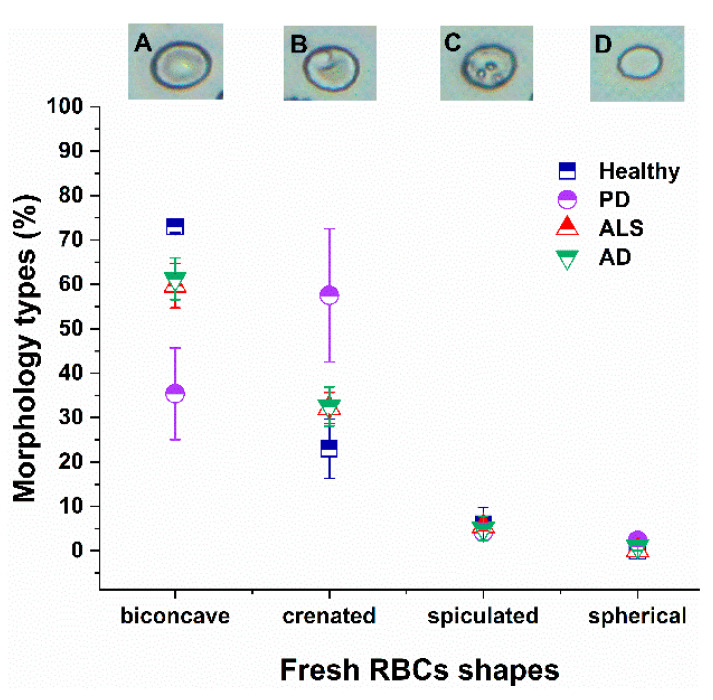
Optical images of the four different RBCs shapes (biconcave (**A**), crenate (**B**), spiculated (**C**) and spherocytic (**D**)). Distribution of RBC morphological types (in percentage) in fresh healthy (navy squares), PD (violet circles), ALS (red triangles) and AD (green inverted triangles) cells. Mean values and SD; *p* values (NDD vs. healthy control group): *p* < 0.01 for biconcave shape of PD, AD and ALS, and for crenate shape of PD cells; and *p* < 0.05 for crenate ALS and AD cells.

The morphology of fresh and aged, healthy and NDDs RBCs was characterized by OM and AFM. OM images of the various morphological types observed along with their distribution in fresh RBCs (isolated on the day of blood sampling) from healthy and NDDs subjects are presented in Figure 1.

Four different cell shapes (biconcave, crenate, spiculated and spherocytic) are distinguished as previously reported for healthy RBCs [47]. The biconcave discoid shape (Figure 1A), characterized by a regular membrane protein network [28], is the dominant shape in fresh healthy cells (ca. 73%). Healthy RBCs also contain ca. 21% crenate shape (Figure 1B) and 6% spiculated (Figure 1C) cell types. The predominant shape of the PD RBCs, however, was the crenate one (54%), followed by the biconcave (36%), and low proportions of spiculated (Figure 1). The morphological composition of both ALS and AD RBCs differed from that of PD cells, and the biconcave shape was most abundant (ca. 60% for ALS and 61% for AD), but reduced relative to the healthy ones, followed by crenate (33% for ALS and AD), and spiculated (5–6% for both ALS and AD) (Figure 1). The contribution of spherocytes is insignificant (less than 2%) in all studied cells. The p-values indicated significant differences for the biconcave and crenate shape of PD, AD and ALS cells.

### 2.2. Aging Pathway of the Morphology of NDDs and Healthy RBCs

**Figure 2:**
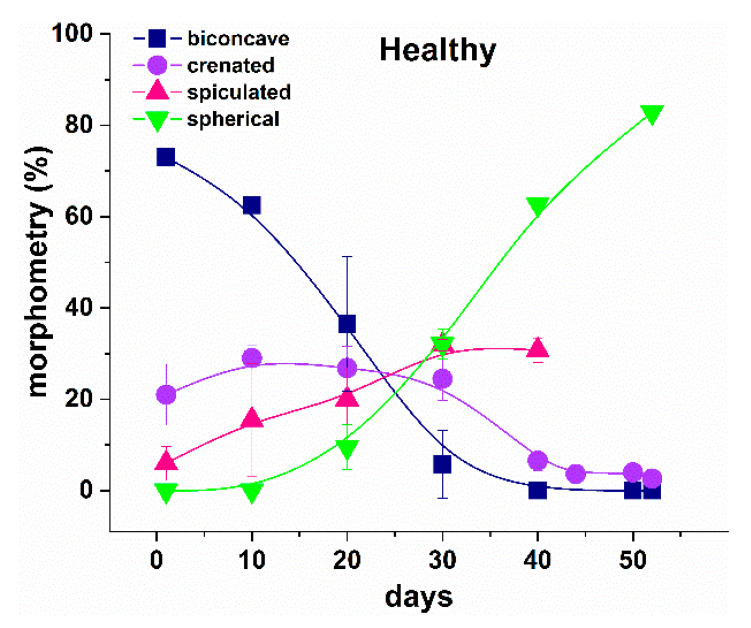
Distribution of the morphological types (in percentage) of healthy RBCs along the aging process, biconcave (navy squares), crenate (violet circles), spiculated (red triangles) and spherocytic (green inverted triangles) shapes. Mean values and SD.

The morphology of healthy RBCs changed during the aging process (Figure 2) as already reported [47]. The main aging effect was associated with a decrease in the proportion of discocytes and crenate shape cells, at the expense of increasing the proportion of the other two morphological types (spicules and spherocytes) along the course of cells aging (Figure 2). At day 20, the biconcave shape was the dominant one, the crenate and spiculated had almost the same contribution, and the spherocytic form had the lowest contribution. Then, at day 30, the biconcave was the lowest fraction, while both spiculated and spherocytic had the highest contribution to the morphological composition of healthy RBCs.

**Figure 3:**
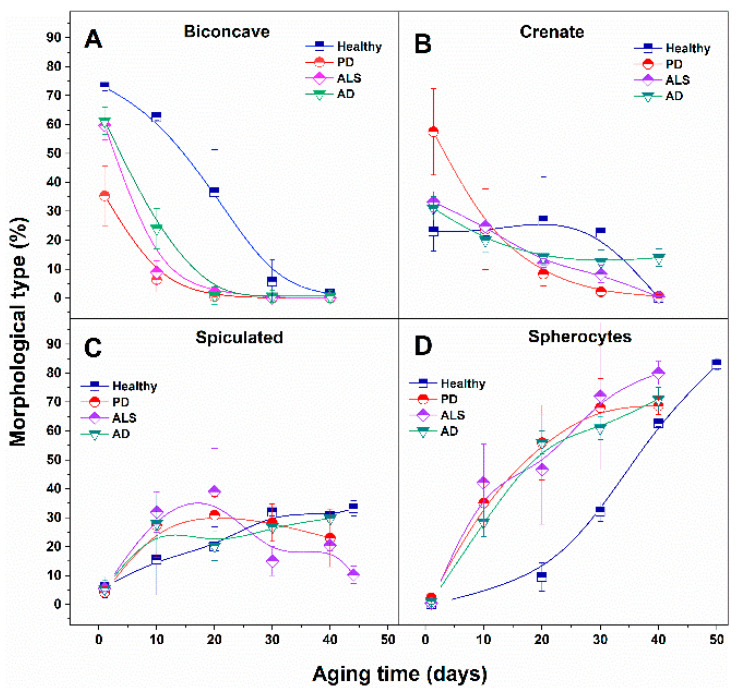
Comparison of the relative contribution (in percentage) of biconcave (**A**), crenate (**B**), speculated (**C**) and spherocytic (**D**) morphological types to healthy, PD, ALS and AD RBCs along the aging time.

A similar trend was revealed not only for healthy RBCs, but also for all NDDs cells studied. However, the transformation of the cell shape occurred with different rates—much faster for the NDDs, compared with the healthy cells. At day 20, the spiculated and spherocytic shapes composed the main fractions for PD, ALS and AD cells, and the biconcave shape was still the highest fraction in the healthy cells (Figure 3).

The abundance of the biconcave type drastically decreased at the 40th day of aging for healthy, and at the 20th day for NDDs cells, respectively. The contribution of the crenated type was not altered significantly along the aging of healthy RBCs up to day 30 and then it was reduced. For ALS and AD cells it was progressively reduced, while for PD cells (for which it was the dominant shape) it drastically decreased as early as day 10, following further the same trend as ALS and AD cells. The reduction in the contribution of crenate cells to the morphology was the weakest for healthy and the most significant for PD cells. The percentage of spiculated and spherocytic cells increased along with the aging process for all studied RBCs. However, the curves of the percentage of cell shape vs. the aging time followed different patterns for healthy and different NDDs cells (Figure 3).

The spiculated irregular shape that had insignificant proportions in fresh cells (from 2.7 to 6%) increased to ca. 30% for 10-day-aged ALS, 20-day-aged PD and 30-day-aged healthy cells (Figure 3). 

The contribution of spherocytes to the morphology of PD, ALS and AD RBCs increased much faster with the aging, reaching 30–40% at day 10, compared to healthy cells (0% at day 10), while their contribution became ca. 70% for PD and AD, ca. 60% healthy cells, and ca. 80% for ALS cells after 40 days of RBCs aging. Hence, in aged RBCs the dominant morphological type present in all studied cells, healthy and NDDs, was spherocytic.

### 2.3. Morphometric Parameters of NDD and Healthy RBCs

Next, we performed more detailed characterization of the morphological parameters (surface roughness, R_rms_; diameter, D; height, H; and volume, V) of healthy and NDDs RBCs by AFM. 

**Figure 4:**
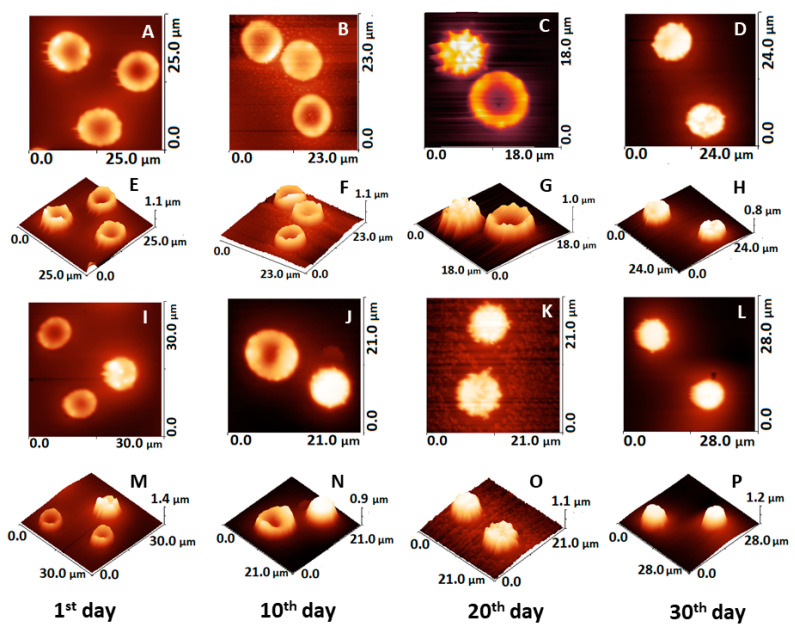
AFM images taken on smears of fresh and aged RBCs from healthy and PD donors on glass support, the scanned area is given on each image. 2D and 3D images of healthy cells: fresh (**A**,**E**), 10-day-aged (**B**,**F**), 20-day-aged (**C**,**G**) and 30-day-aged (**D**,**H**), and of PD cells: fresh (**I**,**M**), 10-day-aged (**J**,**N**), 20-day-aged (**K**,**O**) and 30-day-aged (**L**,**P**).

The 2D and 3D AFM images for healthy and PD cells presented in Figure 4 demonstrated that the various commonly observed shapes in fresh and aged RBCs can be readily distinguished and characterized, as well as their morphology transformation with aging (Figure 4A–D). It is clearly seen that the cell morphology is changed during aging, crenatures and spicules appear in PD cells much earlier than in healthy ones (Figure 4I,J).

**Figure 5:**
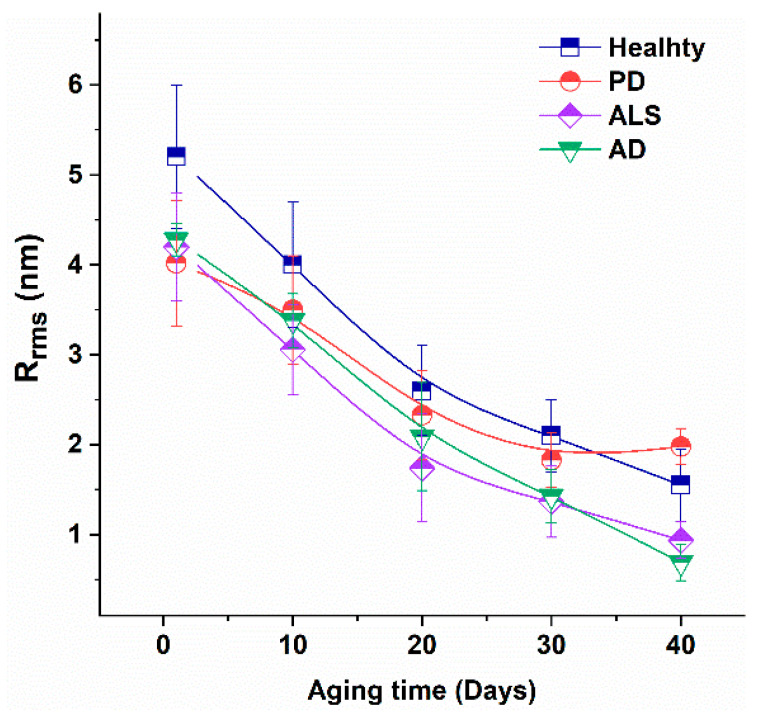
Surface roughness (R_rms_) of healthy, PD, ALS and AD cells vs aging time. Mean values and SD.

The membrane surface roughness, R_rms_, considered to be a measure of the RBC membrane skeleton integrity [47,48,49], had similar values for fresh RBCs derived from patients with the three studied pathologies, smaller than the value for fresh healthy cells (Figure 5). R_rms_ is age-dependent parameter that decreased gradually during the aging process. It is to be noted that the R_rms_ value determined for fresh NDDs cells was equal to that of 10-day-aged healthy cells. 

Therefore, data indicated modified cytoskeletal integrity of NDDs cells compared to healthy ones and stronger effect of aging on surface roughness in NDDs than in healthy cells. The p-values proved the high statistical significance of R_rms_ (*p* = 2 × 10^−7^ for PD and *p* < 0.01 for ALS fresh RBCs). The *p*-value of R_rms_ for ALS cells was kept <0.01 along the aging process, while for PD cells *p* < 0.05 up to day 20, but not statistically significant after this aging period.

**Figure 6:**
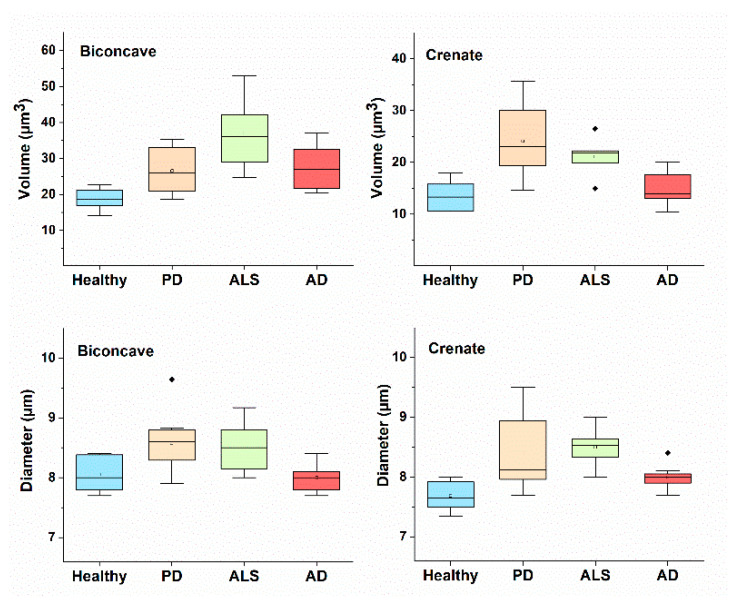
Diameter and volume of biconcave and crenate shape of PD, AD and ALS compared to healthy RBCs. Mean values and SD.

The morphological parameters (D, H and V) for each RBCs’ shape (biconcave, crenate, spiculated and spherocytic) were evaluated for NDDs patients and healthy subjects and summarized in Table 1. Data for the diameter and volume of biconcave and crenate shape cells are presented in Figure 6.

The three morphological parameters—D, H and V, had higher mean values for the biconcave discoids of NDDs, compared to healthy cells (Table 1). It is important to note that for a given health or pathological condition and a given type of RBC shape, the morphological parameters did not change statistically with aging time. Hence, the established differences in the parameters of a certain type of cells are specific for the PD, AD or ALS pathology and could be detected irrespective of the cell age.

The diameter and volume of the biconcave and crenated shapes differed for healthy and NDDs cells, the latter having bigger mean volume as a result of the increased cells diameter (Table 1, Figure 6). The same applies for the volume of the spiculated cells, with exception of AD for which the volume is significantly lower than the volume of PD and ALS. Therefore, crenated and spiculated can be used along with the biconcave type to distinguish the healthy from NDDs RBCs, the latter being more voluminous. PD and ALS spherocytes showed very similar morphological parameters and cannot be distinguished from each other. Considering the statistical analysis, significant differences were found for the volume of biconcave (*p* < 0.05) and both the diameter and the volume (*p* < 0.01) of crenate ALS vs healthy cells, and for the diameter (*p* < 0.05) of biconcave PD vs healthy cells (Table 1). Therefore, the differences in the diameter and volume of crenate ALS RBCs, and the diameter of biconcave PD cells can distinguish ALS and PD from healthy cells.

### 2.4. Young’s Modulus of NDDs and Healthy RBCs

**Figure 7:**
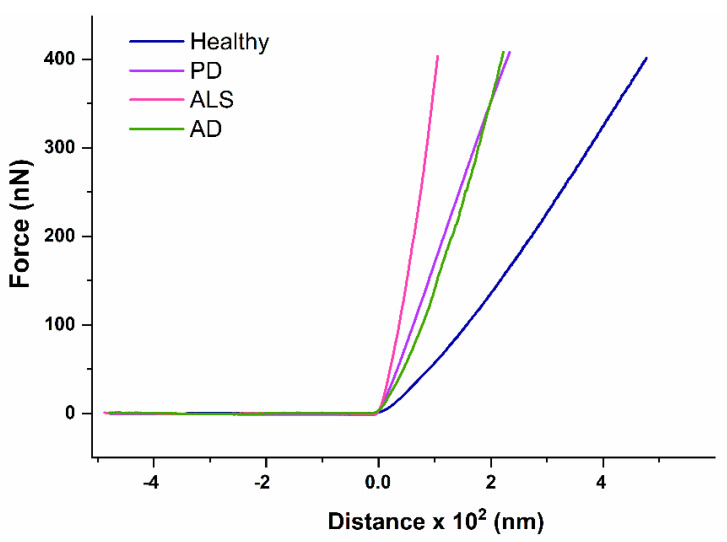
Representative force-distance curves of RBCs derived from healthy individuals and patients diagnosed with PD, AD and ALS.

The Young’s moduli (*E*) were determined from force-distance curves for healthy subjects and NDDs patients (representative curves are shown in Figure 7). 

**Figure 8:**
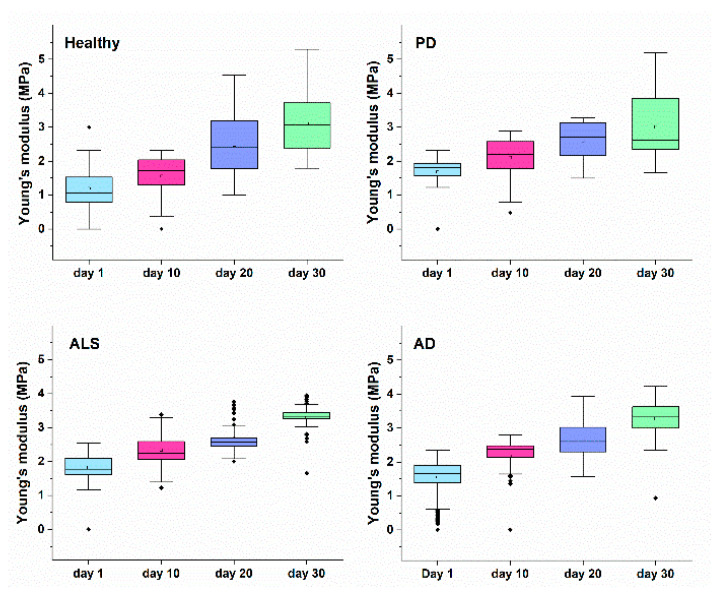
Young’s modulus of healthy, PD, ALS and AD RBCs along the process of cells aging.

*E* reflected the RBCs mechanics, and had different values for healthy and NDDs cells, much lower for the healthy than for the diseased ones (Table 2, Figure 8). Besides, the *E* value increased with cell aging, the difference in *E* between the fresh and aged cells being greater for healthy than for NDDs cells (Δ*E*_aged-fresh_ changed in the following order healthy > AD > ALS > PD). These data indicated that the studied disorders as well as the cells´ aging were associated with increase in the RBCs stiffness. The disease-caused stiffening effect was more significant for fresh ALS compared to PD and AD cells (Figure 8). An approximation of the *E* values of healthy and NDDs RBCs was observed during aging, especially for 20- and 30-day-aged cells.

## 3. Discussion

Different techniques such as AFM, scanning electron microscopy (SEM), OM, optical (laser) tweezers, and microfluidic devices have been explored to characterize human RBCs [47,50,51,52,53,54,55]. It has been reported that a number of pathologies (diabetes mellitus, hereditary disorders (spherocytosis, elliptocytosis), systemic lupus erythematosus (SLE), parasitic disease etc.) are related to modification of RBCs topographical features [28,52,53,55,56,57,58,59,60,61]. AFM and SEM of erythrocytes in diabetic (type 2 diabetes) patients revealed lower values of the diameter, height and surface area, and higher average surface roughness (R_a_) and stiffness compared to healthy cells, as well as irregular elongated shape and a dispersion of large particles on the cell surface versus biconcave shape and small particles on the surface of healthy RBCs [53]. The disease status of diabetes patients was correlated with membrane reorganization [57]. These strong alterations in the diabetic erythrocyte structure and morphology were attributed to the presence of misfolded protein aggregation. It has been demonstrated by meta-analysis of longitudinal studies that patients with diabetes have an increased risk of developing dementia and AD, and the multifactorial mechanism linking these diseases was described in [21]. In the study of Deng et al. [54] significant increase in the surface roughness (average, R_a_, and root mean square, R_rms_) was reported for malformed RBCs from patients during extracorporeal circulation in heart surgery compared with the healthy group. RBCs from patients with SLE display densely arranged circular shaped holes and deformations [52] responsible for the reduced binding of DNA to the erythrocyte surface in SLE patients, as observed by Huss et al. [61]. Exploring AFM based infrared nanospectroscopy (AFM-IR) Ruggeri et al. [62] succeeded in detecting localized oxidative stress and peroxidation of the membrane of biconcave cells at subcellular level, that occurred before morphological changes. 

Moreover, the main physiological function of RBCs to deliver oxygen to tissues depends on cell metabolism, cell-cell interactions, deformability and aggregation, with the cell deformability being the crucial factor [63]. Furthermore, the membrane composition and organization were closely related to cell deformation and aggregation, that is affected by cell morphology, surface properties/membrane surface roughness as well as by the environment [63]. In fact, the cell membrane is responsible for reversible RBC shape deformations that are essential for their function [64] and normally healthy cells have higher deformability than diseased ones [63,65]. 

On the other hand, aging- and pathology-related modifications in membrane organization and reduced tissue oxygenation were associated with decreased deformability, changes in aggregation properties and also in cell shape [66,67]. 

Importantly, statistically significant correlations between deformability and aggregation parameters determined in healthy RBCs were not found in the distorted RBCs of patients with neuroacanthocytosis [63]. This was attributed to disturbed phosphorylation-controlled binding between Band 3 containing membrane protein complexes and the cytoskeleton [31,63,68].

In the present study we have quantitatively assessed the morphological and mechanical features of RBCs from NDDs patients. Our ultrastructural analysis of fresh healthy RBCs revealed predominant contribution of biconcave and presence of crenate shape and insignificant proportion of spicules. The proportion of the typical for healthy RBCs biconcave shape was reduced in NDDs cells, most remarkably in PD cells. The percentage varies in the order healthy > AD ≈ ALS > PD. The crenate type was the predominant morphology of PD RBCs, and has the lowest contribution in healthy cells (PD > ALS > AD > healthy). An equally small percentage of spiculated type, and close to 0% of spherocytes, was found in fresh healthy and NDDs RBCs. Hence, the morphology of NDDs RBCs is distinct from that of healthy cells and can be considered as a signature of these pathologies.

The unique discoid shape of RBCs is maintained by its membrane [64] and can be altered by modifications of the lipid bilayer (as stated in the bilayer-couple hypothesis [69,70,71]), and of the membrane skeleton network supported by vertical (between the lipid bilayer and the membrane skeleton (Band-3, ankyrin, spectrin and band 4.1 proteins)) and horizontal (within the membrane skeleton) interactions [55,72]. On the other hand, the shape of RBCs has been explained in the context of the bending energy hypothesis assuming the difference in bending energy between biconcave and flat configuration area [73]. The biconcave shape, among the variety of shapes RBC can adopt, minimizes the elastic energy stored in the membrane and increases the surface area providing high deformability of the cells. The two-dimensional network of spectrin molecules and the lipid bilayer contribute to the RBC shear elastic properties and shear resistance, respectively [55,64]. Furthermore, fresh healthy biconcave discoids are characterized by high surface-to-volume ratio and large elastic deformability that is extremely important for the RBCs function to transport oxygen and carbon dioxide [55]. The crenate shape has been attributed to the presence of concentrated hemoglobin in the cytosol or to the membrane resistance to bend and can also be described in the framework of the bending hypothesis [74]. Transformation of the shape of RBCs was found to be associated with either increase (from discocytes to echinocytes) or decrease (discocytes to stomatocytes) of the protein area on the extracellular surface [57].

Our results also showed that the morphology of NDDs RBCs experienced age-induced shape transformation and revealed contribution of four morphological types as already reported for normal healthy cells during their aging [47]. Age-dependent changes in RBCs have also been reviewed in [75,76]. 

Importantly, apart from the distinct morphology of fresh NDDs and healthy RBCs, its transformation along the aging of NDDs cells also differs from that of healthy cells. The contribution of the biconcave form to NDDs RBCs decreased much stronger and much faster compared to healthy cells during aging.

The percentage of the crenate morphological type was progressively reduced with the aging time for NDDs cells, while for healthy cells was reduced after day 30. Drastically different aging patterns were found for the spherocytes, which, although being the predominant cell shape at the end of the aging process for all studied cells, demonstrate a contribution that increased with different kinetics—much faster for the three pathologies than for normal healthy state.

Hence, the studied pathologies cause strong alteration in the shape and the morphological composition of NDDs cells that might originate from strongly altered membrane cytoskeleton and/or lipid bilayer as stated in in vivo and in vitro studies of the aging of RBCs [48,52]. The accumulation of toxic misfolded proteins/aggregates in NDDs, shown to circulate in the blood stream and found in RBCs [4,17,43], might affect the cell shape and might account for the observed morphological transformation. Recent in vitro study of human RBC interaction with Aβ has shown accelerated shape alteration, development of crenatures and proto-spicules correlated with weakening of the cell cytoskeleton contacts [77].

As suggested by Girasole et al. [78] one of the mechanisms for the appearance of swollen RBCs is weakening of the cytoskeleton structure. The transformation of the typical biconcave to the spherical form, that is thermodynamically more favorable [79], along RBCs aging might be a result from strong modification of the cytoskeleton interaction network. Conformational change in RBC proteins and redistribution of the membrane phospholipids have been found to correlate with expansion of one layer of the membrane double layer and consequently to shape changes [80,81]. Estimation of the volume of membrane proteins localized on the external membrane surface indicated considerable difference for the different cell shapes [80]. Recent single cell Raman spectra analysis revealed two-step aging of RBCs, the first one related to conformational changes of proteins and the second one to alterations in lipids [82]. 

Our quantitative morphological data prove significant differences between the volume of the disc-shaped cells of NDDs and healthy subjects. The same is true for the crenate and the spiculated PD and ALS, but not for AD cells, that allow for differentiation of AD from PD and ALS RBCs. Therefore, independent of the abundance of the morphological types, the morphological parameters, especially the volume, that characterize each cell shape type differ between NDDs and healthy RBCs. All together the type of the predominant RBCs shape combined with the cell volume can be used as markers for the presence of NDDs. 

Furthermore, we found that the roughness of fresh NDDs cells was much lower than that evaluated for fresh healthy cells, but it was almost equal for PD, ALS and AD cells, which was another evidence for similar modification in the cytoskeleton integrity of NDDs RBCs. Keeping in mind the findings of Girasole et al. [50,51] that the surface roughness is a morphological parameter independent of the shape of RBCs, we can state that the roughness is a common specific feature of NDDs that distinguish them from the normal healthy state. It is to be mentioned that the roughness of RBCs strongly decreased along the aging process, its values were lower for NDDs than for healthy cells indicating that the difference in this parameter between healthy and NDDs was kept stable along the aging. Reduction in the surface roughness of RBCs has already been found for some other pathologies [48,83]. 

Our data also indicated strongly altered membrane stiffness of NDDs RBCs that encipher the erythrocyte shape. The *E* values were higher for NDDs compared to healthy cells, most significantly for ALS cells, suggesting decreased deformability of NDDs relative to healthy cells. These data are consistent with the reported higher *E* moduli for PD and AD patients [32,84]. While for AD patients the higher *E* values were related to high serum ferritin level [84], no such relation has been found for PD patients [32]. The Young’s modulus values of PD patients were recovered to normal ones by treatment with the chelator desferal [32]. Importantly, aging of RBCs exerts similar effect on the membrane stiffness as the neurodegenerative disorders, i.e., increase in *E*. 

Therefore, our results prove common modification of the surface nanostructure, morphometric and nanomechanical features of the studied NDDs relative to healthy RBCs that agrees with accepted view that common pathological mechanisms govern the studied disorders.

## 4. Materials and Methods

### 4.1. Selection of Patients

The research investigation was approved by the ethics committee of the University multiprofile hospital for active treatment in neurology and psychiatry “St. Naum” (UMHATNP), Sofia, (Consent number 05/15.03.2018) and was conducted in agreement with the principles of the Declaration of Helsinki of 1975, revised in 2013 for research involving human subjects. All patients signed an informed consent.

The selected twenty-two patients were diagnosed on clinical criteria with NDDs. Nine PD (4 males and 5 females, mean age 68.2 ± 11.2 (47 to 86 years)) patients involved in the study all fulfilled the 2015 MDS-PD clinical criteria [85]. Patients with comorbid dementia were excluded. The selection of ALS patients was based on the El Escorial criteria [86]. Nine patients with ALS (5 males and 4 females, 59.1 ± 12.2 (42 to 78 years)) were enrolled—6 with clinically definite and 3 with clinically probable and laboratory supported forms of ALS [86]. Four patients with probable AD (4 females, mean age 59.1 ± 12.2 (70 to 83 years)) were selected in the study [87]. 

Control group of 9 healthy individuals (6 females and 3 males, mean age 58.8 ± 10.2 years (42 to 76 years)), none of them is a smoker, has received any treatment, and has a history of any neurodegenerative, hereditary burden or another disease, were included in the study. 

### 4.2. RBCs Preparation

Venous blood samples were taken from the patients and healthy volunteers into EDTA vacutainers (0.084 mL 15% EDTA Becton, Dickinson and Company, Franklin Lakes, NJ, USA). 

RBCs were separated from blood samples by centrifugation at 3000 rpm for 15 min at 4 °C and washed three times with PBS buffer (10 mM sodium phosphate, pH 7.2, 140 mM NaCl, and 1 mM ethylenediaminetetraacetic acid (EDTA) and stored at 4 °C for experiments on cells aging.

Smears of RBCs on glass slides were prepared for OM and AFM measurements according to the protocol described in Dinarelli et al. [47]. 

### 4.3. Optical Microscopy

The morphological types of RBCs from NDDs patients and healthy individuals were determined from the optical images of the cells obtained by light microscope (3D Optical profiler, Zeta-20, Zeta Instruments, Milpitas, CA, USA). About 300 cells for each morphological type were evaluated for statistical reliability. 

### 4.4. Atomic Force Microscopy of RBCs

AFM imaging and force measurements of smears of RBCs were performed with atomic force microscope (MFP-3D, Asylum Research, Oxford Instruments, Abingdon, UK) at room temperature. 

The imaging experiments were carried out in tapping mode in air using standard silicon nitride (Si3N4) probe tips, tip radius < 10 nm (Budget Sensors, Innovative Solutions Ltd., Bulgaria) with a frequency of 16 kHz and a spring constant of 0.06 N/m. 

The AFM images were analyzed using Gwyddion-2.57 software to determine the surface roughness (root-mean-square surface roughness, R_rms_), diameter (D), height (H) and volume (V) of RBCs.

The biconcave discoid and crenate RBCs have a shape approaching that of an elliptic torus. Therefore, the volume of these types of cells was calculated using the formula for the volume of an elliptic torus:

V = 2π^2^*ab*R (1)

where *a* and *b* are the minor and the major radii, respectively, of an ellipse (the major radius is actually the half of the height of RBCs with biconcave or crenate shape), and R is the radius of the revolution of the torus (the distance between the center of the torus and the center of the elliptic cross-section). The values of *a*, *b* and R were determined from the AFM topography images of the RBCs analyzing the profile of each cell. 

In the case of spiculated and spherocytic RBCs, which in shape resemble a thin flat cylinder, the volume was calculated using the formula for the volume of a cylinder using the data for the diameter and the height of the cells, acquired from the AFM topography images.

R_rms_ was evaluated as root-mean-square of the height distribution [48,88] by the equation:

(2) $$R_{\mathrm{rms}}=\sqrt{\sum_{i=1}^{N}\frac{{\left(Zi-Zn\right)}^{2}}{\left(N-1\right)}}$$

where *N* is the total number of data points, *Z_i_* is the height of the ith point and *Z_n_* is the mean height. 

Force-distance curves were measured at room temperature in contact mode using silicon nitride probes (Nanosensors, type qp-Bio) with spring constant of 0.06 N/m, resonant frequency 16 kHz, conical shape and nominal tip radius of 8 nm. The force mapping was performed on a grid of 32 × 32 points. The images were collected at a scanning speed of ca. 2 s/row. The measurements were undertaken on the external ridge of the biconcave and crenate cells, and in the center of spherosites and spicules. Before the measurements the tip was calibrated on a clean glass substrate using special software Igor Pro. 

The Young’s modulus (*E*) was determined by analyzing the force-distance curves applying the Hertz model [89,90].

(3) $$F(\delta )=\frac{2Etan\left(\alpha \right)}{\pi \left(1-\nu^{2}\right)}$$

where *E* corresponds to the apparent Young’s modulus, *υ* is the Poisson ratio and δ is the indentation depth. 

### 4.5. Statistical Analysis 

Statistical analysis of the AFM data was performed by non-parametric statistical test in OriginPro 2018 software. The difference of the R_rms_, D and V, and *E* values of RBCs from NDDs groups vs healthy group was considered statistically significant for *p* < 0.05. 

## 5. Conclusions

This report provides new information on the RBCs ultrastructural changes induced by neurodegenerative disorders. For the first time a direct comparison was made between the morphology and membrane stiffness of RBCs from patients with PD, ALS and AD, and healthy cells.

Data allow us to conclude that: (i) the biconcave disc shape contribution to the cell´s morphology is strongly reduced in NDDs cells, but it is still the dominant shape of ALS and AD cells, while the crenate one is the most abundant in PD cells; (ii) the RBCs´ morphology is age dependent, its transformation follows different aging pathways for NDDs and normal healthy states; (iii) the diameter and volume of NDDs´ biconcave discoid and crenate shape cells have higher values than those of healthy ones; (iv) reduced surface roughness and increased membrane stiffness are characteristic for NDDs cells compared to healthy ones, and both parameters are strongly dependent on cells aging. Our results also show that some specific transformations in the morphology and membrane stiffness observed in fresh NDDs´ RBCs occur in healthy cells later along their aging.

Therefore, we could identify some common hallmarks that distinguish the three NDDs from normal healthy state (R_rms_ values/R_rms_ aging pattern, *E* moduli/*E* moduli values along cells’ aging; D and V values of the different morphological types, biconcave discoid shape contribution to RBCs morphology, aging patterns of biconcave discoids and spherocytes), as well as specific markers: dominant contribution of the crenate morphological type for PD, lower stiffness as well as the highest V of the biconcave discoids and D of crenate cells for ALS cells. These ultrastructural features of RBCs appear to be promising candidates for biomarkers for NDDs and can assist in the diagnosis of the studied pathologies.

## Figures and Tables

**Table 1:** Morphological parameters—diameter (D), height (H) and volume (V) of biconcave, crenate, spiculated and spherocytic shapes of RBCs from healthy subjects and NDDs patients. Mean values and SD.

RBC Shape	Subject	D (µm)	H (µm)	V (µm^3^)
biconcave	Healthy	8.02 ± 0.34	0.57 ± 0.14	18.01 ± 3.59
	PD	8.69 ± 0.50 *	0.58 ± 0.12	25.52 ± 7.27
	ALS	8.57 ± 0.48 *	0.66 ± 0.11	36.79 ± 13.27 *
	AD	8.06 ± 0.24	0.6 ± 0.013	27.35 ± 8.02 *
crenate	Healthy	7.75 ± 0.25	0.50 ± 0.05	13.60 ± 2.94
	PD	8.39 ± 0.93 *	0.51 ± 0.06	24.09 ± 9.97 *
	ALS	8.50 ± 0.37 **	0.54 ± 0.04	21.08 ± 4.20 **
	AD	8.00 ± 0.10	0.51 ± 0.05	15.15 ± 3.0
spiculated	Healthy	6.49 ± 0.27	0.55 ± 0.14	18.05 ± 4.53
	PD	6.67 ± 0.94	0.65 ± 0.10	24.80 ± 3.77
	ALS	6.47 ± 0.49	0.71 ± 0.10	23.37 ± 3.82
	AD	5.83 ± 0.48	0.54 ± 0.12	14.73 ± 4.38
spherocytic	Healthy	6.71 ± 0.33	0.46 ± 0.12	15.86 ± 2.60
	PD	7.30 ± 0.94	0.50 ± 0.08	19.33 ± 1.82
	ALS	7.07 ± 0.12	0.46 ± 0.05	18.22 ± 2.62
	AD	7.77 ± 0.09	0.40 ± 0.04	19.05 ± 1.32

*p* values < 0.05 are denoted by * and *p* < 0.01 by **.

**Table 2:** Young’s modulus (MPa) determined for fresh and aged healthy and NDDs RBCs, mean values ± SD.

	Subject		RBCs	
	Fresh	10-Day-Aged	20-Day-Aged	30-Day-Aged
Healthy	1.18 ± 0.5	1.56 ± 0.6	2.48 ± 0.8	3.10 ± 0.8
PD	1.67 ± 0.4 *	2.10 ± 0.5 *	2.59 ± 0.5	2.99 ± 0.9
ALS	1.82 ± 0.5 *	2.34 ± 0.4 *	2.69 ± 0.4 *	3.29 ± 0.3 *
AD	1.55 ± 0.5	2.18 ± 0.4 *	2.65 ± 0.5	3.28 ± 0.4 *

*p* values < 0.05 are denoted by *.

## Data Availability

All data are included in this work.
